# In-situ child and adolescent mental health simulation with human factors feedback delivered by airline pilots

**DOI:** 10.1192/bjo.2021.388

**Published:** 2021-06-18

**Authors:** Megan Fisher, Alexander Jolly, Mumtaz Mooncey, Kerry Robinson, Robert Lloyd, Dave Fielding

**Affiliations:** 1 Whittington Health NHS Trust; 2 Airline Captain; 3 University College London Hospital

## Abstract

**Aims:**

To encourage multidisciplinary team learning by introducing Child & Adolescent Mental Health (CAMHS) in-situ simulation training.

To provide focused Human Factors feedback through the expertise of senior airline pilots.

**Method:**

The integration of the WingFactors in-situ simulation programme to multiple departments at Whittington Health NHS Trust has transformed the education landscape. The programme has received unanimously positive feedback, and the potential benefits for not only physical, but also mental health training, have been quickly recognised. A total of 90 simulations have been performed. A number of CAMHS scenarios have been designed with the primary aims of encouraging multidisciplinary training and increasing the focus on Human Factors in Psychiatry.

Simulation scenarios were performed in real clinical environments with primed actors, thus enabling high-fidelity in-situ simulation. Immediate ‘hot’ debriefs were delivered by clinical faculty and uniformed airline pilots, with emphasis on psychological safety to encourage participation from all team members. The key learning points were then detailed in written documents and circulated to the wider team as a valuable learning resource.

The first CAMHS simulation involved the acute management of a collapsed patient in the Emergency Department toilet, with a ligature tied around her neck and accompanied by a distressed patient. Another scenario addressed de-escalation techniques when dealing with a patient presenting with an overdose, who was threatening to leave the ward and posing potential risk to herself.

**Result:**

The nature of these in-situ simulations enabled the multidisciplinary team to analyse practical considerations in the management of acute clinical situations. Scenarios were designed to focus on areas which had been identified as needing improvement for patient safety.

The observations provided by airline pilots increased the focus on Human Factors training. A number of key themes were identified, including the importance of effective team-briefing, distraction management and task allocation. This is of particular significance when managing a distressed patient and anxious relative, in a busy high-stress clinical environment.

**Conclusion:**

In-situ simulation is a newly emerging concept in the field of Psychiatry, and the success of this programme has been highlighted through consistently positive feedback from participants, and nomination for the HSJ Award (Best Education Programme 2021). The involvement of airline pilots has promoted collaborative learning amongst the multidisciplinary team, and increased the focus on Human Factors in Psychiatry, clearly demonstrating the value of in-situ simulation training in this field.

